# Control of membrane gaps by synaptotagmin-Ca^2+^ measured with a novel membrane distance ruler

**DOI:** 10.1038/ncomms6859

**Published:** 2014-12-15

**Authors:** Chao-Chen Lin, Jan Seikowski, Angel Pérez-Lara, Reinhard Jahn, Claudia Höbartner, Peter Jomo Walla

**Affiliations:** 1 Research Group Biomolecular Spectroscopy and Single-Molecule Detection, Max Planck Institute for Biophysical Chemistry, Am Faßberg 11, 37077 Göttingen, Germany; 2 Research Group Nucleic Acid Chemistry, Max Planck Institute for Biophysical Chemistry, Am Faßberg 11, 37077 Göttingen, Germany; 3 Department of Neurobiology, Max Planck Institute for Biophysical Chemistry, Am Faßberg 11, 37077 Göttingen, Germany; 4 Institute for Organic and Biomolecular Chemistry, Georg August University Göttingen, Tammannstraße 2, 37077 Göttingen, Germany; 5 Department of Biophysical Chemistry, Institute for Physical and Theoretical Chemistry, University of Braunschweig, Hans-Sommer-Straße 10, 38106 Braunschweig, Germany

## Abstract

Fast synchronous neurotransmitter release is triggered by calcium that activates synaptotagmin-1 (syt-1), resulting in fusion of synaptic vesicles with the presynaptic membrane. Syt-1 possesses two Ca^2+^-binding C2 domains that tether membranes via interactions with anionic phospholipids. It is capable of crosslinking membranes and has recently been speculated to trigger fusion by decreasing the gap between them. As quantitative information on membrane gaps is key to understanding general cellular mechanisms, including the role of syt-1, we developed a fluorescence-lifetime based inter-membrane distance ruler using membrane-anchored DNAs of various lengths as calibration standards. Wild-type and mutant data provide evidence that full-length syt-1 indeed regulates membrane gaps: without Ca^2+^, syt-1 maintains membranes at distances of ~7–8 nm. Activation with 100 μM Ca^2+^ decreases the distance to ~5 nm by binding the C2 domains to opposing membranes, respectively. These values reveal that activated syt-1 adjusts membrane distances to the level that promotes SNARE complex assembly.

## Abstract (web summary)

Synaptotagmin-1 (syt-1) triggers fast synchronous neurotransmission mediated by fusion of synaptic vesicles with presynaptic membranes. Here Lin *et al.* use inter-membrane FRET combined with DNA rulers as calibration standards to quantify Ca^2+^-induced changes in membrane distances mediated by syt-1.

## Graphical abstract

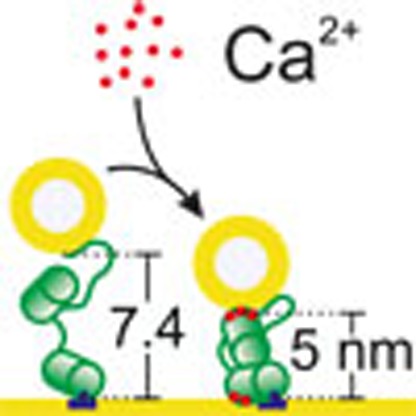

Neurotransmission is accomplished by exocytosis following the fusion of synaptic vesicles with the presynaptic plasma membrane^1^,^2^,^3^. The neurotransmitters stored within the vesicles are released into the synaptic cleft at a millisecond timescale on Ca^2+^ influx^4^, with syt-1, residing on the synaptic vesicles and bearing two Ca^2+^-binding C2 domains (C2A and C2B), being the indispensable Ca^2+^ sensor^5^,^6^ (Fig. 1). The C2A and C2B domains, after coordinating to multiple Ca^2+^ ions^7^,^8^,^9^, bind to membranes containing negatively charged phospholipids, which complete the Ca^2+^-coordination sites^8^. Moreover, a patch of four lysine residues (KKKK, 324–327) located on the side of C2B interacts specifically with the poly-anionic phosphatidylinositol(4,5)-bisphosphate (PIP2) in a Ca^2+^-independent manner^10^. However, despite extensive research using either the full-length syt-1 (including the transmembrane domain, TMD) or the truncated soluble C2AB fragment^11^,^12^,^13^, the questions how syt-1 functions at a molecular level as a Ca^2+^-trigger and how it interacts with the SNARE proteins (soluble *N*-ethylmaleimide-sensitive factor attachment protein receptor), the membrane fusion machinery^1^,^2^, remained unsettled.

Before the formation of fully zippered SNARE complexes, which exert forces on the membranes and lead consequently to fusion, the synaptic vesicles are first primed to the presynaptic plasma membrane and await the Ca^2+^ activation of syt-1. The models describing the molecular activation of the fusion reaction by synaptotagmin fall into two groups (summarized in ref. 14). In the first scenario, the SNARE complexes are arrested at a partially zippered state during the priming process. Syt-1 may either serve as a fusion clamp that is released on Ca^2+^ triggering^15^, or alternatively, it may displace the inhibitory protein complexin from the SNARE complexes^16^. In the second scenario, the SNAREs do not assemble before arrival of the Ca^2+^ signal, although syt-1 may already be in contact with the plasma membrane via the KKKK–PIP2 electrostatic interaction^10^. Following Ca^2+^ triggering, the SNAREs rapidly progress through zippering and fusion^17^.

In line with the latter scenario, recently, it has been proposed that syt-1 triggering is based on Ca^2+^-dependent regulation of the gap between the vesicle and presynaptic membranes. In the absence of Ca^2+^, syt-1 connects membranes via (i) its own transmembrane domain and (ii) binding to PIP2 via the KKKK polybasic stretch. Assuming that the 61-residue linker^18^ between C2A and the transmembrane domain is unstructured and stretched, a maximal distance of ~28 nm is feasible. On the basis of non-quantitative Förster resonance energy transfer (FRET) between labelled lipids in the participating membranes, it was suggested that addition of Ca^2+^ might reduce the distance to a range of ~2–7.5 nm (ref. 19). Such distance shortening might operate upstream of the SNARE complex formation, which requires a membrane distance below 8 nm (ref. 20). However, experimental support for this model was still elusive, primarily because of the lack of experimental evidence for distance variations at the appropriate length scale.

In this study, we first developed a quantitative membrane distance assay based on accurate fluorescence lifetime detection of multiple inter-membrane FRET interactions between tethered small unilamellar liposomes using membrane-anchored double-stranded DNA (dsDNA) of defined lengths as calibration standards. This calibrated system was then used to measure membrane distances of liposomes tethered by full-length syt-1 wild-type as well as Ca^2+^-binding mutants in the absence and presence of physiological Ca^2+^ concentrations and when the content of anionic lipids required for syt-1 binding was varied. These data provide evidence that Ca^2+^-activated syt-1 indeed decreases membrane gaps to a level of ~5 nm, which corresponds to the inter-membrane distance spanned by a *trans-*SNARE complex.

## Results

### Membrane distance ruler

**Figure 2:**
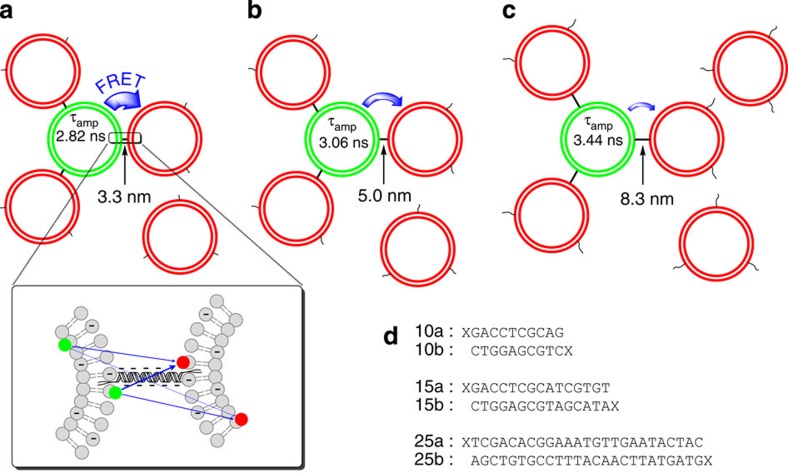
FRET from green donor liposomes to the surrounding red acceptor liposomes. The upper panels show the experimental design of the membrane distance ruler for small unilamellar liposomes, with (**a**) 3.3, (**b**) 5.0 and (**c**) 8.3 nm spacings. FRET decreases as the distance increases, as reflected in the longer amplitude-weighted lifetime (*τ*_amp_). (**d**) The complementary DNA sequences for the respective lengths, where X denotes the lipid-phosphoramidite.

The fluorescence lifetime of donor molecules labelled on lipid headgroups (Oregon Green 488 DHPE) of the DNA- or proteoliposomes was chosen as a robust indicator of FRET, as it is insensitive to variations in the concentration of liposomes within the confocal volume of ~1 femtoliter or to variations in alignment conditions of the experimental set-up. In contrast to FRET-based distance measurements within or between interacting proteins^21^,^22^,^23^, distances between membranes cannot be easily extracted. In proteins, single donor and acceptor dyes are attached to defined sites and a single donor–acceptor pair is distant from the other fluorophores. In these cases, the average distance between the pair can be easily derived, providing that the Förster distance *R*_0_ for the selected donor and acceptor is known. On liposomes, contrarily, the fluorophores are distributed across the surfaces of both the inner and outer leaflets. Hence, in a single tethered cluster, each donor molecule may transfer the excitation energy to multiple acceptors, and the probability of each donor undergoing FRET is different and depends on its position relative to the acceptor liposomes containing Texas Red DHPE (Fig. 2a), which in turn leads to different lifetimes. Therefore, we measured first a calibration curve using tethered liposomes with predefined spacings so that the average donor fluorescence lifetimes can be correlated properly with membrane distances between donor and acceptor liposomes. The calibration of this membrane distance ruler was done using membrane-anchored dsDNA of various well-defined lengths as discrete spacers^24^. In general, dsDNA shorter than its persistence length of 50 nm, or 150 base pairs (bp), is known to have high bending rigidity^25^. Herein three different lengths well below the persistence length were prepared (Fig. 2), 3.3 nm (10 bp), 5.0 nm (15 bp) and 8.3 nm (25bp). The membrane anchor lipid 1,2-*O*-dioctadecyl-(rac)-glycerol was attached to the 5′-end of DNA oligonucleotides by solid-phase synthesis using a synthetic lipid phosphoramidite^26^. The complementary strands were then individually reconstituted into the donor and acceptor liposomes, respectively. In addition, 10% anionic lipids phosphatidylserine (PS) were included in both types of DNA liposomes. The negative charges on the liposome surfaces and the negatively charged DNA backbone further stabilized the perpendicular orientation of the DNA already observed in the absence of any repulsive charges on the membranes^27^.

An excess of acceptor liposomes was used to drive the equilibrium so that each donor liposome is surrounded by an equal (maximal) number of acceptor liposomes (Fig. 2a–c), to eliminate the contribution of differences in the degree of tethering^28^. To determine the required ratio, we gradually increased the acceptor liposome concentration while keeping the donor liposomes constant. The donor fluorescence decay curves converged after the ratio exceeded 1:10 (Supplementary Fig. 1). Therefore, a ratio of 20 acceptor liposomes to 1 donor liposome was selected for all experiments.

**Figure 3:**
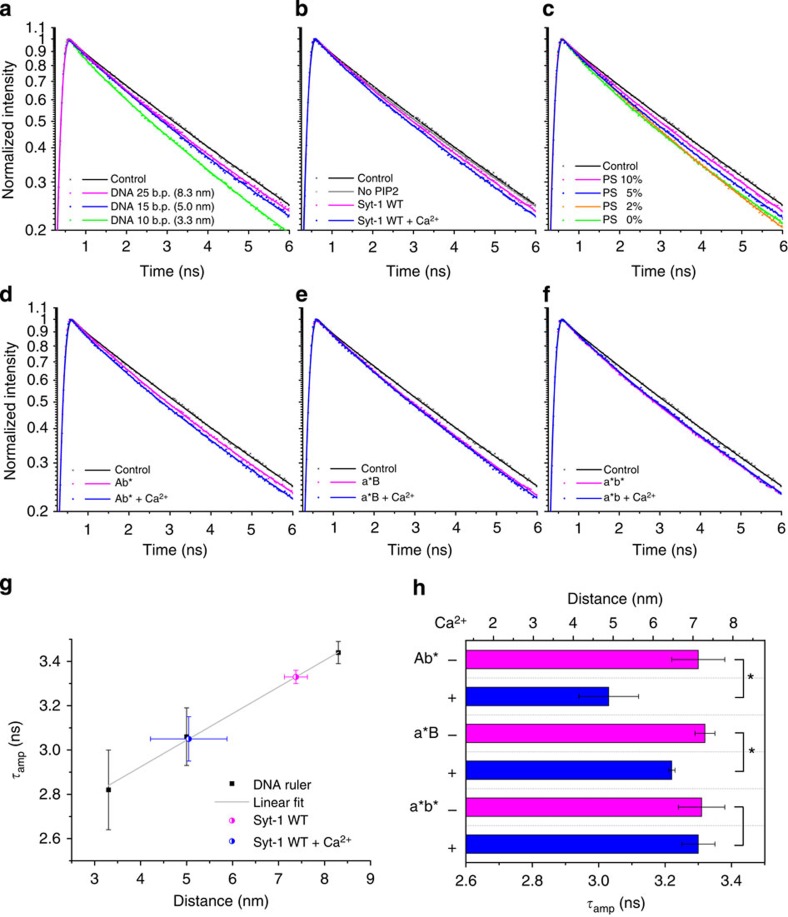
The measured fluorescence decay curves and the membrane distance ruler for converting fitted *τ*_amp_ into closest distances between small unilamellar liposomes. Decay curves for (**a**) DNA-tethered liposomes with specified number of base pairs and (**b**) syt-1 wild-type (WT) reconstituted into donor liposomes in the absence and presence of 100 μM Ca^2+^. No PIP2 indicates the sample in which 2% PIP2 was substituted with 7% PS. (**c**) Measurements without Ca^2+^ for syt-1 WT reconstituted into donor liposomes with various molar ratios of the anionic lipid PS. (**d**–**f**) Decay curves for Ab*, a*B and a*b* mutants. Control stands for the experiments in which there was no protein or DNA on either type of liposomes. The symbols represent experimental data, whereas the lines are reconvolution fits using two exponentials. (**g**) The plot of amplitude-weighted lifetime (*τ*_amp_) versus distance. A linear regression line can be constructed with the DNA-tethered liposomes and applied to determine the distance between syt-1 tethered liposomes in the absence or presence of 100 μM Ca^2+^. (**h**) *τ*_amp_ and the calculated distance information for the mutants. The error bars represent s.d. values obtained from three independent repeats. Asterisks (*) designate when the *τ*_amp_ values are statistically different (*P*<0.05, two-tailed unpaired *t*-test).

The fluorescence decay curves of the three DNA-tethered liposome mixtures were measured with time-correlated single photon counting^29^ (TCSPC) (Fig. 3a), and the emission wavelength was selected at the blue edge of donor fluorescence spectrum (525±10 nm) to minimize the crosstalk between donor and acceptor channels (Supplementary Fig. 2). The three curves were clearly distinguishable and decayed faster than the control sample without DNA, and the difference arises predominantly from the donor molecules that are facing the acceptor liposomes. The decay curves observed with the aforementioned excess ratio of 1:20 were fitted with two components, and the amplitude-weighted lifetimes (*τ*_amp_, see Methods section) of the donors depending on inter-membrane distances were obtained. By measuring three independent replicates, *τ*_amp_ for liposomes with membrane distances of 8.3, 5.0 and 3.3 nm was determined to be 3.44±0.05, 3.06±0.13 and 2.82±0.18 ns, respectively (average±s.d.). A plot of *τ*_amp_ versus the membrane distance can be very well fitted by a linear approximation (Fig. 3g), which later serves as the calibration curve for membrane distance determination. As control, hybridization of complementary DNA strands on the liposomes was inhibited by adding a free single strand (without the lipid anchor, Supplementary Fig. 3). Under these conditions, the lifetime was restored to the control level observed with a sample containing donor and excess acceptor liposomes, neither with DNA. An advantage of the calibration procedure (Fig. 3g) is that any nonspecific effects, such as nonspecific liposome clustering, are intrinsically corrected for. To quantify the extent of such nonspecific effects, we compared a sample containing only pure donor liposomes (grey dashed line in Supplementary Fig. 3) directly with the donor–acceptor control without any DNA (black line in Supplementary Fig. 3). This comparison demonstrates that nonspecific effects contribute only to a minor extent to the overall fluorescence decay.

It is known from electron microscopy (EM) studies that small unilamellar liposomes (≤50 nm), owing to their high membrane curvature, still maintain the spherical shape at contact areas when tethered by proteins (see for example, cryo-electron tomography in ref. 30). To ensure additionally that the number of membrane tethering sites between donor and acceptor liposomes are comparable in all samples, we conducted fluorescence correlation spectroscopy (FCS, see Supplementary Fig. 4 and Supplementary Methods for details). FCS data showed that the average number of acceptor liposome tethering sites per donor liposome did not vary by >15% throughout all samples measured in this work (Supplementary Table 1).

### Synaptotagmin-1 controls the gap between two membranes

Next, we reconstituted syt-1 in donor liposomes containing 10% PS and measured the decay when they were bound to acceptor liposomes bearing 15% PS plus 2% PIP2 (Fig. 3b)^3^,^31^. In the absence of Ca^2+^ (1 mM EGTA), *τ*_amp_ was close to that of the 8.3 nm DNA-tethered liposomes (3.33±0.03 ns) and the membrane distance was estimated to be 7.4 nm (Fig. 3b,g). To verify that the decrease in fluorescence lifetime compared with the control sample was caused by the specific interaction of the KKKK patch with PIP2, the poly-anionic PIP2 (2%) in the acceptor liposomes was replaced by increasing the concentration of mono-anionic PS by 7 to 22% PS, which maintained the net negative charge of the acceptor liposomes^32^. In the absence of PIP2, the fluorescence lifetime returned to the control level, showing that tethering to the syt-1 proteoliposomes was abolished. We then repeated the incubation using PIP2-containing liposomes in the absence of Ca^2+^ (1 mM EGTA) to allow for the KKKK-PIP2 binding and added appropriate Ca^2+^ buffer afterwards to achieve a final free Ca^2+^ concentration of 100 μM. The resulting fluorescence decay was accelerated and corresponded to that measured with liposomes tethered with the DNA-ruler at a distance of 5.0 nm (*τ*_amp_=3.05±0.10) (Fig. 3b,g). We conclude accordingly that the gap between syt-1 proteoliposomes and the surrounding acceptor liposomes decreases to ~5 nm after the addition of Ca^2+^.

The distance of 7.4 nm in the absence of Ca^2+^ is relatively short, suggesting that the linker between the transmembrane domain and the C2A domain is not completely stretched^19^. Intriguingly, the linker contains a membrane-adjacent stretch of positive charges, which may interact with the more C-terminally localized stretch of negative charges^33^ and/or with anionic lipids in the resident membrane of the protein (a *cis* interaction)^34^. To further examine the electrostatic interactions with negative charges on the liposome membranes^28^, we performed a set of experiments in which the content of PS on the donor proteoliposomes was varied (Fig. 3c). When the donor proteoliposomes were neutral, that is, without PS, and only the acceptor liposomes contained anionic lipids, the measured lifetime was surprisingly short (2.82±0.09 ns) even in the absence of Ca^2+^ and was similar to that of the shortest set of DNA-tethered liposomes (3.3 nm). This short distance can be explained by the nonphysiological elimination of electrostatic repulsion between the two membranes, which is present *in vivo* between the negatively charged synaptic vesicle and presynaptic membranes^3^,^31^. In addition, the membranes may be pulled into close proximity if the positively charged lysine residues on the linker are attached in *trans* to the surfaces of acceptor liposomes. Figure 3c also indicates that at an intermediate level of PS (5%), there is an equilibrium between the 7.4- and 3.3-nm configurations.

### Ca^2+^-binding mutants

As all known Ca^2+^ effects are mediated by the Ca^2+^-binding sites in the C2A and C2B domains, we repeated the distance measurements using previously characterized syt-1 mutants in which Ca^2+^ binding to the C2A, C2B or to both C2 domains was disrupted (a*B: D178A, D230A and D232A; Ab*: D309A, D363A and D365A; a*b*: D178A, D230A, D232A, D309A, D363A and D365A)^11^. In the mutant with a disrupted C2B domain (Ab*), the inter-membrane distances in the absence and presence of Ca^2+^ were very similar to those of the wild-type protein (Fig. 3d,g,h). In contrast, in the mutants with a disrupted C2A domain (a*B and a*b*), there was little or no distance shortening on addition of Ca^2+^, respectively (Fig. 3e,f,h). Summarized in Fig. 3h are the fitted *τ*_amp_ and the corresponding distances calculated for all mutants. These data support a model according to which the C2A domain is predominantly responsible for distance shortening due to Ca^2+^-mediated *cis* binding to its own membrane. On the contrary, the C2B domain, at least under our experimental conditions, does not appear to be capable of simultaneous *cis*-*trans* binding as previously suggested^8^,^35^. The fact that a*B still exhibits a minor decrease in distance on Ca^2+^ addition may rather be explained by the Ca^2+^-induced binding of C2B to the acceptor membrane and the insertion of its hydrophobic residues^10^,^36^.

## Discussion

**Figure 4:**
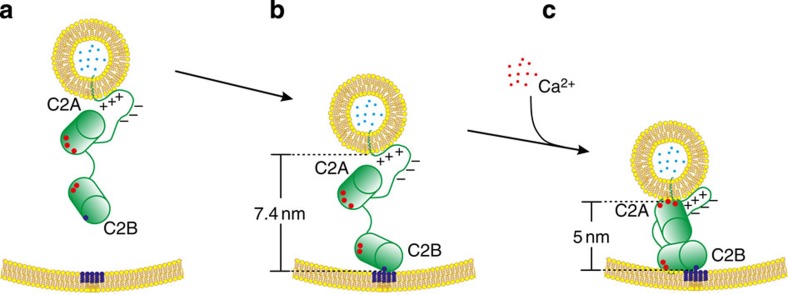
Model of full-length syt-1 binding across the synaptic vesicle and the presynaptic plasma membrane. (**a**) Syt-1 targets PIP2 clusters via the poly-lysine patch (blue). (**b**) The distance between two membranes is maintained at ~7-8 nm, as the linker is not fully stretched^34^. (**c**) On Ca^2+^ influx, the C2A domain binds *cis*, reducing the distance to 5 nm and potentially promoting the electrostatic zippering of the linker^33^. Both membranes contain PS.

The calibrated fluorescence-lifetime-based membrane distance ruler with membrane-anchored DNAs of well-defined lengths allowed for direct quantitative determination of membrane gaps controlled by wild-type and mutants of the full-length neurotransmission trigger syt-1. Initially, in the absence of Ca^2+^, the poly-lysine patch (KKKK) located on the C2B domain is targeted to PIP2 clusters on the presynaptic plasma membrane (Fig. 4a,b)^37^,^38^. Our data demonstrate that at this stage full-length syt-1 maintains the two membrane bilayers at a distance of ~7–8 nm (Fig. 4b), which is much shorter than a maximum length of ~28 nm if the linker would be unstructured and fully stretched. The ~7–8 nm distance corresponds to that when the SNARE motifs start to assemble and form the coiled coil four-helix bundle (measured with surface forces apparatus)^20^, implying that syt-1 holds the two membranes at this distance so that the fusion machinery is ready for fast initiation^17^. Recently, such a pre-triggered state was also captured in cryo-EM images in ref. 30, which revealed that a large number of small liposomes incorporating syt-1 and synaptobrevin (the *vesicle*-SNARE protein) remained docked to giant liposomes including syntaxin and SNAP-25 (the *target*-SNAREs) at distances on the order of ~10 nm, even after extended incubation. The fact that removing synaptobrevin or adding its soluble counterpart to inhibit the full-length SNARE assembly did not alter the overall tethering suggests the major role of syt-1 in establishing a first contact to the target membrane, upstream of SNARE nucleation (Fig. 4b)^19^,^39^.

Second, the ruler provides clear evidence that, on Ca^2+^ influx, the inter-membrane gap is compressed to ~5 nm (Fig. 4c). As a *trans-*SNARE complex spans two membranes at a distance of ≥4 nm (ref. 40), a distance reduction from ~7–8 nm to ~5 nm brings the two membranes to a level at which very likely full assembly of the SNAREs is promoted^41^, leading subsequently to the initiation of membrane fusion and neurotransmitter release. At first glance, the ~2.4 nm change in distance may not seem very large in terms of the absolute value. However, considering the above-mentioned dimensions of the SNAREs, such a change is quite significant and allows for efficient triggering at the millisecond timescale.

The membrane distance of 5 nm also correlates to cryo-EM observations^35^,^42^ for liposomes clustered by soluble C2AB fragments incorporating Ca^2+^, in which the C2A and C2B domains bind to opposing membranes^43^. Such an antiparallel conformation has been shown to be of the lowest energy from simulated annealing based on EPR-derived restraints using the soluble C2AB fragment^44^,^45^,^46^. Moreover, it corroborates new findings that alteration in the length and rigidity of the short (9-residue) linker between the C2 domains has a significant impact on evoked neurotransmitter release^47^.

*In vivo* mutation studies demonstrated that the C2B domain is indispensable for fast synchronous neurotransmitter release^48^,^49^. The fact that the distance regulation behaviour of the Ab* mutant is similar to that of the wild-type (Fig. 3g,h) illustrates that in contrast to the C2B domain—which plays an essential general role—C2A plays a more important role in the distance regulation itself: initially, the C2B domain is needed to tether the membranes together, especially via its poly-lysine patch also in the absence of Ca^2+^ (Fig. 4b). After C2B is already attached to the opposite membrane, an effective decrease of the inter-membrane gap can then be accomplished by binding of the C2A domain back to the host membrane and an even tighter binding of the C2B domain to the opposite membrane with Ca^2+^ coordination (Fig. 4c). Obviously, the role of C2B Ca^2+^ binding sites is less connected to the distance regulation function of syt-1 than to other important mechanistic functions, which might include direct interactions with the SNAREs^15^,^21^,^23^,^50^ or facilitating curvature of the presynaptic membrane^51^,^52^. Notably, recently an ~80% decrease in release has been demonstrated using a D229E mutation in *Drosophila* at the Ca^2+^-coordination site of C2A^53^, supporting the important function also of C2A in synchronous synaptic transmission.

As a final remark, the membrane distance ruler developed in this study using membrane-anchored DNAs will also be very beneficial to the investigation of distance regulatory steps in other biological processes of interest, such as fertilization, viral entry as well as protein trafficking^54^,^55^, in which the gap between two membranes needs to be quantified at the nm-scale.

## Methods

### Sample preparation

Syt-1 (Rat) wild-type and the mutants were purified as previously described^28^ and elaborated in Supplementary Methods. The DNA-lipid conjugates were synthesized by solid-phase synthesis using the lipid-phosphoramidite^26^ as the last base under otherwise standard DNA synthesis conditions^56^ and purified by RP-HPLC (see Supplementary Methods for details and characterization). Small unilammelar liposomes were prepared using Sephadex G-50 superfine (Sigma-Aldrich) size-exclusion columns. The size of the resulting liposomes was ~40 nm in diameter, estimated from the FCS information recorded simultaneously^57^ and dynamic light scattering (DLS). Liposomes were labelled either with 0.5% OG-DHPE or with 1% TR-DHPE (Invitrogen), and the DNA/protein-to-lipid ratios were kept at 1:1,000. The proteoliposomes consisted of 20% DOPE, 10% cholesterol and 10% DOPS, whereas the acceptor liposomes contained 20% DOPE, 10% cholesterol, 15% DOPS and 2% PIP2, unless otherwise specified. Ten percent DOPS was included into all DNA liposomes. The remaining lipids were adjusted with DOPC to yield 100%. All non-labelled lipids were purchased from Avanti Polar Lipids. The buffer contained 150 mM KCl, 20 mM Hepes and was adjusted to pH 7.4. Hybridization of the DNA-liposomes was achieved by incubation for 5 min at 35 °C for 10 bp and 55 °C for 15 and 25 bp, followed by slow cooling back to room temperature. Syt-1 proteoliposomes were first incubated with the acceptor liposomes for 30 min and then added to buffers containing 1 mM EGTA or a final Ca^2+^ concentration of 100 μM (checked with Fluo-5N, Invitrogen) for another 30 min to reach equilibrium. Final concentrations of donor liposomes were maintained at ~2 nM (one particle in the focal volume on average).

### Instrumentation

Fluorescence microscopy measurements were performed on an Olympus IX71 inverted microscope with an UPlanSApo × 60/1.2 W water immersion objective. A Ti:sapphire oscillator (Chameleon, Coherent) centering at 800 nm served as the two-photon excitation source. A dichroic mirror 715DCSPXR (AHF) and a short pass filter E700SP were used to separate the excitation and emission and the dichroic mirror 590DCXR (AHF) to split the fluorescence onto the green and red avalanche photodiode detectors (SPCM-CD 2969, PerkinElmer), selected with band pass filters D525/20m and D680/30m (Chroma). Data were acquired with a router (PRT 400)-coupled TCSPC card (TimeHarp200, PicoQuant) and analysed with the SymPhoTime software version 5.3 (PicoQuant).

### Amplitude-weighted lifetime (*τ*_amp_)

For numerical determination of the distances, we fitted all the fluorescence decay curves with iterative reconvolution using a model function of two exponential components: *I*(*t*)=*A*_1_exp(−*t*/*τ*_1_)+*A*_2_exp(−*t*/*τ*_2_). The amplitude-weighted lifetime (*τ*_amp_=*a*_1_*τ*_1_+*a*_2_*τ*_2_, where *a*_1_=*A*_1_/(*A*_1_+*A*_2_) and *a*_2_=*A*_2_/(*A*_1_+*A*_2_)), which is more sensitive to changes in the faster component, was then calculated.

## Author contributions

C.-C.L., R.J. and P.J.W. wrote the paper. J.S. and C.H. synthesized the DNA lipids. A.P.-L. and R.J. provided the proteins. C.-C.L. designed the study and performed all other experiments. All authors discussed the results and commented on the manuscript.

## Additional information

**How to cite this article:** Lin, C.-C. *et al.* Control of membrane gaps by synaptotagmin-Ca^2+^ measured with a novel membrane distance ruler. *Nat. Commun.* 5:5859 doi: 10.1038/ncomms6859 (2014).

## Supplementary Material

Supplementary Information Supplementary Figure 1-4, Supplementary Table 1, Supplementary Methods and Supplementary References

## Figures and Tables

**Figure 1:**
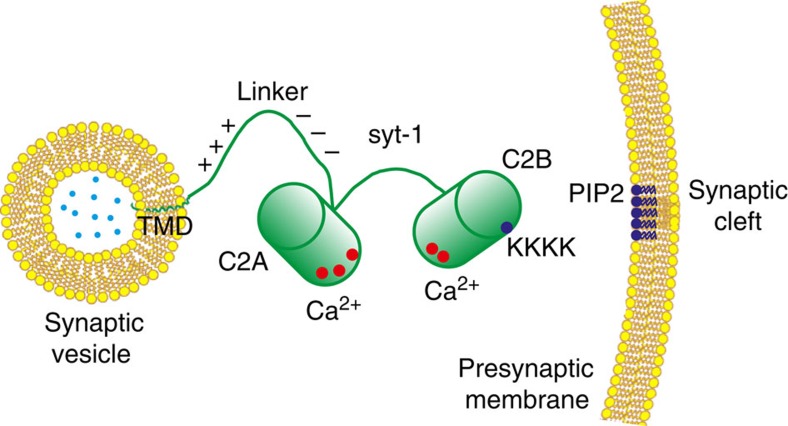
Schematic illustration of the synaptotagmin-1 domain structure. Red dots indicate Ca^2+^-binding sites of the two Ca^2+^-binding domains C2A and C2B, whereas the dark blue dot represents the poly-lysine patch (KKKK) of the C2B domain. Plus and minus signs indicate clusters of positive and negative charges on the linker. Bright blue dots represent neurotransmitter molecules. PIP2, phosphatidylinositol(4,5)-bisphosphate; TMD, transmembrane domain.

## References

[b1] Jahn R. & Südhof T. C. Membrane fusion and exocytosis. Annu. Rev. Biochem. 68, 863–911 (1999). 10872468 10.1146/annurev.biochem.68.1.863

[b2] Jahn R. & Scheller R. H. SNAREs−engines for membrane fusion. Nat. Rev. Mol. Cell Biol. 7, 631–643 (2006). 16912714 10.1038/nrm2002

[b3] Takamori S. *et al.* Molecular anatomy of a trafficking organelle. Cell 127, 831–846 (2006). 17110340 10.1016/j.cell.2006.10.030

[b4] Schneggenburger R. & Neher E. Intracellular calcium dependence of transmitter release rates at a fast central synapse. Nature 406, 889–893 (2000). 10972290 10.1038/35022702

[b5] Chapman E. R. How does synaptotagmin trigger neurotransmitter release? Annu. Rev. Biochem. 77, 615–641 (2008). 18275379 10.1146/annurev.biochem.77.062005.101135

[b6] Brose N., Petrenko A. G., Südhof T. C. & Jahn R. Synaptotagmin: a calcium sensor on the synaptic vesicle surface. Science 256, 1021–1025 (1992). 1589771 10.1126/science.1589771

[b7] Ubach J., Zhang X., Shao X., Südhof T. C. & Rizo J. Ca^2+^ binding to synaptotagmin: how many Ca^2+^ ions bind to the tip of a C2‐domain? EMBO J. 17, 3921–3930 (1998). 9670009 10.1093/emboj/17.14.3921 PMC1170727

[b8] Honigmann A. *et al.* Phosphatidylinositol 4,5-bisphosphate clusters act as molecular beacons for vesicle recruitment. Nat. Struct. Mol. Biol. 20, 679–686 (2013). 23665582 10.1038/nsmb.2570 PMC3676452

[b9] Fernandez I. *et al.* Three-dimensional structure of the synaptotagmin 1 C2B-domain: synaptotagmin 1 as a phospholipid binding machine. Neuron 32, 1057–1069 (2001). 11754837 10.1016/s0896-6273(01)00548-7

[b10] Bai J., Tucker W. C. & Chapman E. R. PIP_2_ increases the speed of response of synaptotagmin and steers its membrane-penetration activity toward the plasma membrane. Nat. Struct. Mol. Biol. 11, 36–44 (2004). 14718921 10.1038/nsmb709

[b11] Stein A., Radhakrishnan A., Riedel D., Fasshauer D. & Jahn R. Synaptotagmin activates membrane fusion through a Ca^2+^-dependent *trans* interaction with phospholipids. Nat. Struct. Mol. Biol. 14, 904–911 (2007). 17891149 10.1038/nsmb1305

[b12] Wang Z., Liu H., Gu Y. & Chapman E. R. Reconstituted synaptotagmin I mediates vesicle docking, priming, and fusion. J. Cell Biol. 195, 1159–1170 (2011). 22184197 10.1083/jcb.201104079 PMC3246889

[b13] Lee H. K. *et al.* Dynamic Ca^2+^-dependent stimulation of vesicle fusion by membrane-anchored synaptotagmin 1. Science 328, 760–763 (2010). 20448186 10.1126/science.1187722 PMC2994549

[b14] Jahn R. & Fasshauer D. Molecular machines governing exocytosis of synaptic vesicles. Nature 490, 201–207 (2012). 23060190 10.1038/nature11320 PMC4461657

[b15] Chicka M. C., Hui E., Liu H. & Chapman E. R. Synaptotagmin arrests the SNARE complex before triggering fast, efficient membrane fusion in response to Ca^2+^. Nat. Struct. Mol. Biol. 15, 827–835 (2008). 18622390 10.1038/nsmb.1463 PMC2570314

[b16] Yang X., Kaeser-Woo Y. J., Pang Z. P., Xu W. & Südhof T. C. Complexin clamps asynchronous release by blocking a secondary Ca^2+^ sensor via its accessory alpha helix. Neuron 68, 907–920 (2010). 21145004 10.1016/j.neuron.2010.11.001 PMC3050570

[b17] Pobbati A. V., Stein A. & Fasshauer D. N- to C-terminal SNARE complex assembly promotes rapid membrane fusion. Science 313, 673–676 (2006). 16888141 10.1126/science.1129486

[b18] Perin M. S., Brose N., Jahn R. & Südhof T. C. Domain structure of synaptotagmin (p65). J. Biol. Chem. 266, 623–629 (1991). 1985919

[b19] van den Bogaart G. *et al.* Synaptotagmin-1 may be a distance regulator acting upstream of SNARE nucleation. Nat. Struct. Mol. Biol. 18, 805–812 (2011). 21642968 10.1038/nsmb.2061 PMC3130798

[b20] Li F. *et al.* Energetics and dynamics of SNAREpin folding across lipid bilayers. Nat. Struct. Mol. Biol. 14, 890–896 (2007). 17906638 10.1038/nsmb1310

[b21] Vrljic M. *et al.* Molecular mechanism of the synaptotagmin-SNARE interaction in Ca^2+^-triggered vesicle fusion. Nat. Struct. Mol. Biol. 17, 325–331 (2010). 20173762 10.1038/nsmb.1764 PMC2928146

[b22] Brunger A. T., Weninger K., Bowen M. & Chu S. Single-molecule studies of the neuronal SNARE fusion machinery. Annu. Rev. Biochem. 78, 903–928 (2009). 19489736 10.1146/annurev.biochem.77.070306.103621 PMC2854664

[b23] Choi U. B. *et al.* Single-molecule FRET-derived model of the synaptotagmin 1-SNARE fusion complex. Nat. Struct. Mol. Biol. 17, 318–324 (2010). 20173763 10.1038/nsmb.1763 PMC2922927

[b24] Chung M., Koo B. J. & Boxer S. G. Formation and analysis of topographical domains between lipid membranes tethered by DNA hybrids of different lengths. Faraday Discuss. 161, 333–345 (2013). 23805748 10.1039/c2fd20108a PMC3703934

[b25] Baumann C. G., Smith S. B., Bloomfield V. A. & Bustamante C. Ionic effects on the elasticity of single DNA molecules. Proc. Natl Acad. Sci. USA 94, 6185–6190 (1997). 9177192 10.1073/pnas.94.12.6185 PMC21024

[b26] Chan Y. H., van Lengerich B. & Boxer S. G. Lipid-anchored DNA mediates vesicle fusion as observed by lipid and content mixing. Biointerphases 3, FA17–FA21 (2008). 20408664 10.1116/1.2889062

[b27] Chung M., Lowe R. D., Chan Y. H., Ganesan P. V. & Boxer S. G. DNA-tethered membranes formed by giant vesicle rupture. J. Struct. Biol. 168, 190–199 (2009). 19560541 10.1016/j.jsb.2009.06.015 PMC2757119

[b28] Vennekate W. *et al.* *Cis-* and *trans-*membrane interactions of synaptotagmin-1. Proc. Natl Acad. Sci. USA 109, 11037–11042 (2012). 22711810 10.1073/pnas.1116326109 PMC3390864

[b29] Lakowicz J. R. Principles of Fluorescence Spectroscopy 3 edn Springer Science+Business Media, LLC (2006).

[b30] Bharat T. A. *et al.* SNARE and regulatory proteins induce local membrane protrusions to prime docked vesicles for fast calcium-triggered fusion. EMBO Rep. 15, 308–314 (2014). 24493260 10.1002/embr.201337807 PMC3989697

[b31] Smith C. U. M. Elements of Molecular Neurobiology 3 edn John Wiley & Sons, Ltd. (2002).

[b32] McLaughlin S., Wang J., Gambhir A. & Murray D. PIP_2_ and proteins: interactions, organization, and information flow. Annu. Rev. Biophys. Biomol. Struct. 31, 151–175 (2002). 11988466 10.1146/annurev.biophys.31.082901.134259

[b33] Lai Y., Lou X., Jho Y., Yoon T. Y. & Shin Y. K. The synaptotagmin 1 linker may function as an electrostatic zipper that opens for docking but closes for fusion pore opening. Biochem. J. 456, 25–33 (2013). 24001110 10.1042/BJ20130949 PMC4418238

[b34] Lu B., Kiessling V., Tamm L. K. & Cafiso D. S. The juxtamembrane linker of full-length synaptotagmin 1 controls oligomerization and calcium-dependent membrane binding. J. Biol. Chem. 289, 22161–22171 (2014). 24973220 10.1074/jbc.M114.569327 PMC4139229

[b35] Araç D. *et al.* Close membrane-membrane proximity induced by Ca^2+^-dependent multivalent binding of synaptotagmin-1 to phospholipids. Nat. Struct. Mol. Biol. 13, 209–217 (2006). 16491093 10.1038/nsmb1056

[b36] Hui E., Bai J. & Chapman E. R. Ca^2+^-triggered simultaneous membrane penetration of the tandem C2-domains of synaptotagmin I. Biophys. J. 91, 1767–1777 (2006). 16782782 10.1529/biophysj.105.080325 PMC1544279

[b37] van den Bogaart G. *et al.* Membrane protein sequestering by ionic protein-lipid interactions. Nature 479, 552–555 (2011). 22020284 10.1038/nature10545 PMC3409895

[b38] Aoyagi K. *et al.* The activation of exocytotic sites by the formation of phosphatidylinositol 4,5-bisphosphate microdomains at syntaxin clusters. J. Biol. Chem. 280, 17346–17352 (2005). 15741173 10.1074/jbc.M413307200

[b39] Ellena J. F. *et al.* Dynamic structure of lipid-bound synaptobrevin suggests a nucleation-propagation mechanism for trans-SNARE complex formation. Proc. Natl Acad. Sci. USA 106, 20306–20311 (2009). 19918058 10.1073/pnas.0908317106 PMC2787132

[b40] Hanson P. I., Roth R., Morisaki H., Jahn R. & Heuser J. E. Structure and conformational changes in NSF and its membrane receptor complexes visualized by quick-freeze/deep-etch electron microscopy. Cell 90, 523–535 (1997). 9267032 10.1016/s0092-8674(00)80512-7

[b41] Gao Y. *et al.* Single reconstituted neuronal SNARE complexes zipper in three distinct stages. Science 337, 1340–1343 (2012). 22903523 10.1126/science.1224492 PMC3677750

[b42] Seven A. B., Brewer K. D., Shi L., Jiang Q. X. & Rizo J. Prevalent mechanism of membrane bridging by synaptotagmin-1. Proc. Natl Acad. Sci. USA 110, E3243–E3252 (2013). 23918375 10.1073/pnas.1310327110 PMC3752263

[b43] Connell E. *et al.* Cross-linking of phospholipid membranes is a conserved property of calcium-sensitive synaptotagmins. J. Mol. Biol. 380, 42–50 (2008). 18508081 10.1016/j.jmb.2008.01.084 PMC2726287

[b44] Kuo W., Herrick D. Z. & Cafiso D. S. Phosphatidylinositol 4,5-bisphosphate alters synaptotagmin 1 membrane docking and drives opposing bilayers closer together. Biochemistry 50, 2633–2641 (2011). 21344950 10.1021/bi200049c PMC3071796

[b45] Herrick D. Z. *et al.* Solution and membrane-bound conformations of the tandem C2A and C2B domains of synaptotagmin 1: Evidence for bilayer bridging. J. Mol. Biol. 390, 913–923 (2009). 19501597 10.1016/j.jmb.2009.06.007 PMC2763419

[b46] Lai A. L., Huang H., Herrick D. Z., Epp N. & Cafiso D. S. Synaptotagmin 1 and SNAREs form a complex that is structurally heterogeneous. J. Mol. Biol. 405, 696–706 (2011). 21087613 10.1016/j.jmb.2010.11.015 PMC3039131

[b47] Liu H. *et al.* Linker mutations reveal the complexity of synaptotagmin 1 action during synaptic transmission. Nat. Neurosci. 17, 670–677 (2014). 24657966 10.1038/nn.3681 PMC4139111

[b48] Mackler J. M., Drummond J. A., Loewen C. A., Robinson I. M. & Reist N. E. The C2B Ca^2+^-binding motif of synaptotagmin is required for synaptic transmission in vivo. Nature 418, 340–344 (2002). 12110842 10.1038/nature00846

[b49] Nishiki T. & Augustine G. J. Dual roles of the C2B domain of synaptotagmin I in synchronizing Ca^2+^-dependent neurotransmitter release. J. Neurosci. 24, 8542–8550 (2004). 15456828 10.1523/JNEUROSCI.2545-04.2004 PMC6729890

[b50] Bhalla A., Chicka M. C., Tucker W. C. & Chapman E. R. Ca^2+^-synaptotagmin directly regulates t-SNARE function during reconstituted membrane fusion. Nat. Struct. Mol. Biol 13, 323–330 (2006). 16565726 10.1038/nsmb1076

[b51] Martens S., Kozlov M. M. & McMahon H. T. How synaptotagmin promotes membrane fusion. Science 316, 1205–1208 (2007). 17478680 10.1126/science.1142614

[b52] Hui E., Johnson C. P., Yao J., Dunning F. M. & Chapman E. R. Synaptotagmin-mediated bending of the target membrane is a critical step in Ca^2+^-regulated fusion. Cell 138, 709–721 (2009). 19703397 10.1016/j.cell.2009.05.049 PMC2758036

[b53] Striegel A. R. *et al.* Calcium binding by synaptotagmin's C2A domain is an essential element of the electrostatic switch that triggers synchronous synaptic transmission. J. Neurosci. 32, 1253–1260 (2012). 22279210 10.1523/JNEUROSCI.4652-11.2012 PMC3567453

[b54] Martens S. & McMahon H. T. Mechanisms of membrane fusion: disparate players and common principles. Nat. Rev. Mol. Cell Biol. 9, 543–556 (2008). 18496517 10.1038/nrm2417

[b55] Jahn R., Lang T. & Südhof T. C. Membrane Fusion. Cell 112, 519–533 (2003). 12600315 10.1016/s0092-8674(03)00112-0

[b56] Wachowius F., Javadi-Zarnaghi F. & Höbartner C. Combinatorial mutation interference analysis reveals functional nucleotides required for DNA catalysis. Angew. Chem. Int. Ed. 49, 8504–8508 (2010). 10.1002/anie.201003940 20872387

[b57] Cypionka A. *et al.* Discrimination between docking and fusion of liposomes reconstituted with neuronal SNARE-proteins using FCS. Proc. Natl Acad. Sci. USA 106, 18575–18580 (2009). 19843696 10.1073/pnas.0906677106 PMC2764736

