# Supplementary material for: Control of membrane gaps by synaptotagmin-Ca2+ measured with a novel membrane distance ruler
Source: Nat Commun. 2014 Dec 15;5:5859. doi: 10.1038/ncomms6859 (PMC4275583; doi:10.1038/ncomms6859)
Supplement: Supplementary Information — Supplementary Figure 1-4, Supplementary Table 1, Supplementary Methods and Supplementary References [file ncomms6859-s1.pdf]

Supplementary Figures and Tables

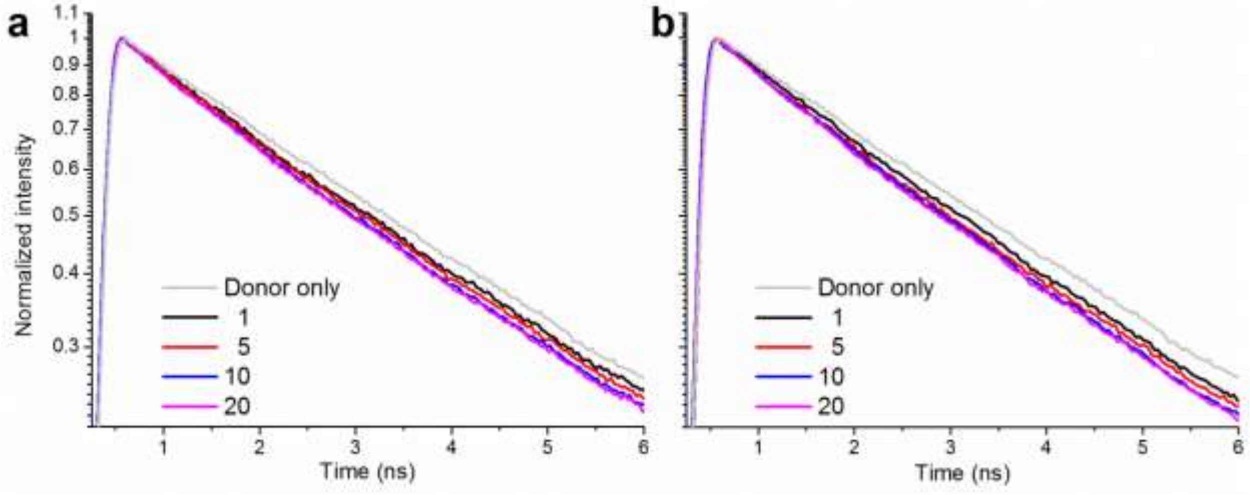

Supplementary Figure 1 | Convergence of the fluorescence decay curves at higher excess ratios. Shown for (**a**) Syt-1 samples in 1 mM EGTA and (**b**) in 100 μM Ca^{2+}. The numbers indicate the ratio of acceptor liposomes relative to the donor liposomes.

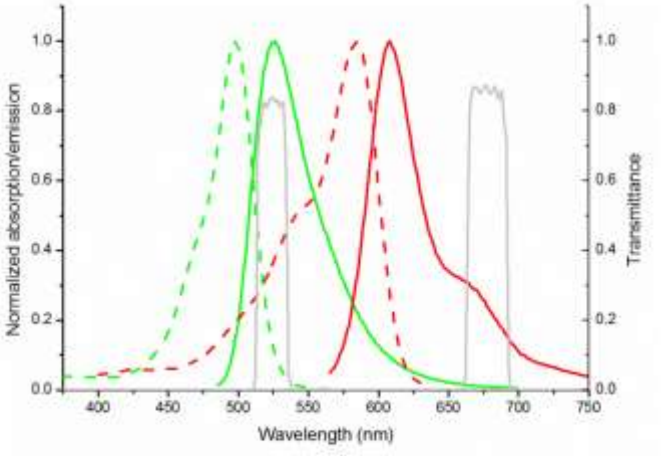

Supplementary Figure 2 | Spectra of the fluorophores and band pass filters. Spectra of the FRET donor, Oregon Green 488 are shown in green and those of the acceptor, Texas Red, in red. The dashed lines are the absorption and solid lines the fluorescence emission. The gray solid lines indicate the transmittance of band pass filters D525/20m and D680/30m. In addition to minimizing cross-talks, monitoring at the blue edge of donor fluorescence selectively amplifies the contribution of donor molecules facing the acceptor liposomes. The amplification arises from the slow nanosecond solvent relaxation processes on membrane surfaces[1]. Donor molecules away from the acceptors decay with longer lifetimes and will experience the relaxation so that the emission is red-shifted out of the blue observation window, losing a larger portion of photons. Conversely, all photons from shorter-lived donors (undergoing more efficient FRET) are collected.

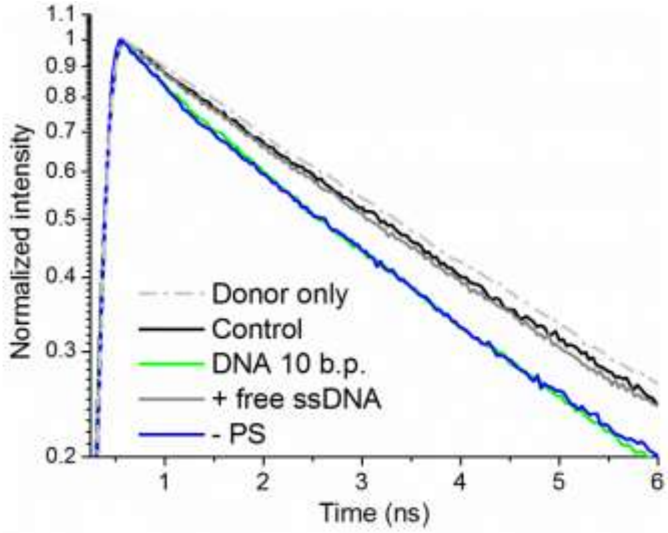

Supplementary Figure 3 | Inhibition of DNA-liposomes hybridization via the addition of a free single strand. Shown for the 10 b.p. samples. The additional blue curve represents the sample in which the negative charges on both types of liposomes were omitted.

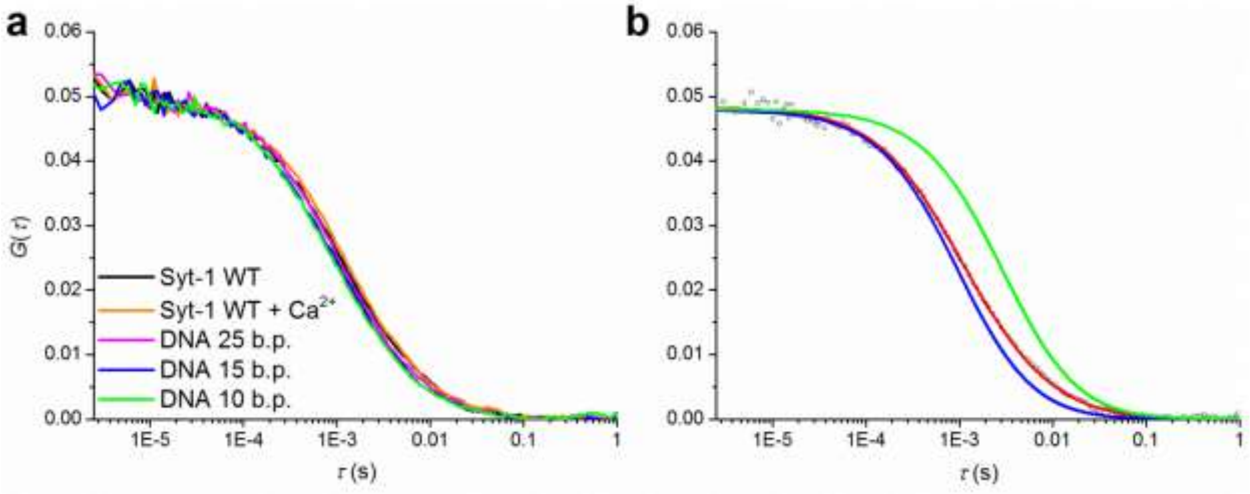

Supplementary Figure 4 | Autocorrelation curves of the acceptor signals and the 2-component fitting. (**a**) Normalized autocorrelation of syt-1 wild-type (WT) and DNA-tethered liposomes at the 1:20 excess ratio. (**b**) Representative 2-component fitting of the syt-1 sample without Ca^{2+}. (◦) The experimental data. Two diffusive components with diffusion times of 1 ms (−) and 3 ms (−) were input as the initial values and (−) is the final 2-component fit.

Syt-1 (EGTA)	Syt-1 (Ca^{2+})	DNA 10 b.p.	DNA 15 b.p.	DNA 25 b.p.
2.9 ± 0.23	3.0 ± 0.19	3.3 ± 0.10	2.6 ± 0.12	2.8 ± 0.18

Supplementary Table 1 | The number of acceptor liposomes bound to each donor liposome determined from 2-component fitting of the autocorrelation curves. Averages and standard deviations were taken from three independent repeats.

Supplementary Methods

Protein constructs and purification

The full-length syt-1 wild-type and Ca^{2+}-binding mutant constructs were cloned into the pET28a vector using NdeI/XhoI restriction sites and expressed in the *E. coli* strain BL21 (DE3). The proteins were purified using Ni^{2+}-NTA affinity chromatography (GE Healthcare), followed by ion-exchange chromatography on the Äkta system (GE Healthcare) in the presence of 1% CHAPS.

Synthesis of lipid-anchored DNA oligonucleotides

The 1,2-*O*-dioctadecyl-(rac)-glycerol 3-(2-cyanoethyl) *N*,*N*-diisopropylphosphoramidite was prepared as described, using 1,2-*O*-dioctadecyl-(rac)-glycerol and 2-cyanoethyl *N*,*N*-diisopropylchlorophosphoramidite in the presence of Hünig's base in dichloromethane[2]. The phosphoramidite was dissolved in anhydrous dichloromethane (100 mM) and used for solid-phase DNA synthesis under otherwise standard conditions[3]. The coupling time for the modified phosphoramidite was 12 min. The DNA oligonucleotides were deprotected and cleaved from the solid support with ammonia in water/ethanol (3:1, 55°C, 20 h), and purified by RP-HPLC on a Resource 15RPC column (1 mL), using a linear gradient of acetonitrile in triethyl ammonium acetate buffer (0-100% in 20 column volumes). Purity and identity of the synthetic DNA was confirmed by HPLC and ESI-MS analysis.

	**5'-Sequence-3'** X=1,2-dioctadecylglycerol	**Mol. Wt. calculated** [g/mol]	**Mol. Wt. found** [g/mol]
10a	XGACCTCGCAG	3671.8	3671.5
10b	XCTGCGAGGTC	3702.8	3703.3
15a	XGACCTCGCATCGTGT	5202.9	5203.3
15b	XATACGATGCGAGGTC	5275.8	5276.2
25a	XTCGACACGGAAATGTTGAATACTAC	8333.0	8333.4
25b	XGTAGTATTCAACATTTCCGTGTCGA	8306.0	8306.8

FCS analysis

The autocorrelation functions of the acceptor fluorescence fluctuations were fitted with 3D diffusion of two species[4]:

$$G(\tau) = \frac{N_1 B_1^2 D_1(\tau) + N_2 B_2^2 D_2(\tau)}{(N_1 B_1 + N_2 B_2)^2} \quad (1)$$

in which N_i and B_i stand for average particle numbers and the brightness of each species, while $D_i(\tau)$ is the diffusion term given by:

$$D_i(\tau) = \frac{1}{1+\tau/\tau_{Di}} \cdot \frac{1}{\sqrt{1+(r_0/z_0)^2 \cdot \tau/\tau_{Di}}} \quad (2)$$

where τ_{Di} denotes the characteristic diffusion times and r_0 and z_0 the dimensions of the effective focal volume.

Fitting of the ACFs was performed with the QuickFit 3.0 software[5]. The τ_{D1} values were determined by measuring the diffusion of free acceptor liposomes and were typically around 1 ms. From the best fits the fractions of the respective species, ρ_1 and ρ_2, were obtained, and the ratio of the fractions can be expressed as:

$$\frac{\rho_1}{\rho_2} = \frac{N_1 B_1^2}{N_2 B_2^2} \quad (3)$$

When the average number of donor liposomes in the focal volume is 1 and that of the acceptor liposomes is N (e.g. 20), the formula can be rearranged to:

$$\frac{N_1}{N_2} = \frac{\rho_1}{\rho_2} \cdot \alpha^2 = N - \alpha \quad (4)$$

with $\alpha = B_2/B_1$ representing also the number of acceptor liposomes bound to a central donor liposome under N times excess. Solving equation (4) yields:

$$\alpha = \frac{-1+\sqrt{1+4N\rho_1/\rho_2}}{2\rho_1/\rho_2} \quad (5)$$

Supplementary References

1. Barucha-Kraszewska, J., Kraszewski, S., Jurkiewicz, P., Ramseyer, C. & Hof, M. Numerical studies of the membrane fluorescent dyes dynamics in ground and excited states. *Biochim. Biophys. Acta-Biomembranes* **1798**, 1724-1734 (2010).
2. Chan, Y. H., van Lengerich, B. & Boxer, S. G. Lipid-anchored DNA mediates vesicle fusion as observed by lipid and content mixing. *Biointerphases* **3**, FA17-FA21 (2008).
3. Wachowius, F., Javadi-Zarnaghi, F. & Höbartner, C. Combinatorial mutation interference analysis reveals functional nucleotides required for DNA catalysis. *Angew. Chem. Int. Ed.* **49**, 8504-8508 (2010).
4. Lakowicz, J. R. *Principles of Fluorescence Spectroscopy*. 3 edn, (Springer Science+Business Media, LLC, 2006).
5. Krieger, J. W. & Langowski, J. (2011): **QuickFit 3.0 (status: beta, compiled: 10.12.2012, SVN: 1959): A data evaluation application for biophysics**, *http://www.dkfz.de/ Macromol/quickfit/*
